# Societal cost-effectiveness analysis of the 21-gene assay in estrogen-receptor–positive, lymph-node–negative early-stage breast cancer in Japan

**DOI:** 10.1186/1472-6963-14-372

**Published:** 2014-09-05

**Authors:** Hideko Yamauchi, Chizuko Nakagawa, Shinji Yamashige, Hiroyuki Takei, Hiroshi Yagata, Atsushi Yoshida, Naoki Hayashi, John Hornberger, Tiffany Yu, Calvin Chao, Carl Yoshizawa, Seigo Nakamura

**Affiliations:** Department of Breast Surgery, St. Luke’s International Hospitalcph, Tokyo, Japan; Graduate School of Economics, Hitotsubashi University, Tokyo, Japan; Division of Breast Surgery, Saitama Cancer Center, Saitama, Japan; Cedar Associates LLC, Menlo Park, CA USA; School of Medicine, Stanford University, Stanford, CA USA; Genomic Health, Inc, Redwood City, CA USA; Department of Breast Surgery, Showa University, Tokyo, Japan

**Keywords:** Breast cancer, Cost-effectiveness, Cost-benefit, Molecular diagnostic testing, Genetic testing

## Abstract

**Background:**

Breast-cancer incidence and mortality have been increasing in Japan. Japanese-specific clinical validity and utility data for the 21-gene assay (Onco*type* DX® Breast Cancer Assay; Genomic Health, Inc., Redwood City, USA) are now available. The objective of this study was to evaluate the cost-effectiveness of the 21-gene assay for the guidance of adjuvant chemotherapy decisions in estrogen-receptor–positive, lymph-node–negative, early-stage breast cancer patients, from the Japanese societal perspective.

**Methods:**

The recurrence risk group distribution by the 21-gene assay result and the assay’s influence on adjuvant chemotherapy recommendations were obtained from a study of 104 patients. A state-transition cohort (Markov) model tracked time from surgery until distant recurrence and from distant recurrence to death. Adjuvant chemotherapy benefit by 21-gene assay risk group was based on published clinical validation studies. Direct and indirect medical costs were obtained from the referral centers. Utilities associated with progression and chemotherapy-related adverse events were extracted from literature. Sensitivity analyses assessed the key drivers and robustness of the primary outcomes.

**Results:**

The 21-gene assay identified 48% of patients as low-risk, 36% as intermediate-risk, and 16% as high-risk. Total acute chemotherapy-related costs decreased by ¥154,066 due to less adjuvant chemotherapy usage. In the high-risk group, adjuvant chemotherapy use increased 18%, leading to survival benefits. Chemotherapy use overall decreased by 19%. Monitoring costs increased by ¥3,744 but recurrence costs declined by ¥46,113 per patient. Use of the 21-gene assay increased quality-adjusted–life-years (QALYs) by 0.241 per patient on average; the net cost per QALY gained was ¥636,752 ($6,368).

**Conclusions:**

The 21-gene assay for women with estrogen-receptor–positive, lymph-node–negative, early-stage breast cancer is projected to be cost-effective in Japan.

## Background

Breast cancer is the most common cancer among Japanese women, with approximately 40,000 women diagnosed every year [1–3]. Although the breast-cancer–specific mortality rate is declining in the United States and other Western countries, it is rising in Japan [4]. At diagnosis, at least 83% of breast cancer in Japan present with early-stage disease (stages 0, I, II, or IIIa) [5, 6]; more than 60% have no lymph node involvement (LN-), and 74% have estrogen-receptor–positive (ER+) breast cancer.

Guidelines issued by the National Comprehensive Cancer Network (NCCN) and the expert panel at the 2011 St. Gallen International Breast Cancer Conference indicate that patients with ER+, LN- early-stage breast cancer (ESBC) have the options of systemic adjuvant treatment with either endocrine therapy and/or chemotherapy [7, 8]. They recommend that clinicians consider selecting candidates for adjuvant chemotherapy to prevent distant recurrence on the basis of clinical and pathologic features, such as patient age, tumor size, degree of lymph node involvement, and tumor differentiation. The potential risk reduction in distant recurrence must be weighed against the risks of adverse events with adjuvant chemotherapy; at least 10% of patients experience serious or life-threatening adverse effects with chemotherapy treatment [9].

The 21-gene assay (Onco*type* DX® Breast Cancer Assay; Genomic Health, Inc., Redwood City, USA) has been shown to predict local and distant recurrence risk, survival, and chemotherapy benefit in ER+ ESBC [10–16]. The 21-gene assay has been commercially available in Japan since 2007. Two observational studies published in 2010 support the validation of the assay in Japan as a strong predictor of distant recurrence risk [3, 17]. Toi et al. conducted a prospective analysis of previously archived tumor samples from 200 women who had ER+, LN- ESBC and had undergone tamoxifen treatment at eight high-volume centers throughout Japan [17]. The distant recurrence rate at 10 years among Japanese women with ER+, LN- ESBC was 9.6% on average, but as high as 24.8% in the high-risk group. Yorozuya et al. conducted a retrospective, case–control study in 40 patients who had surgery for ER+, LN- ESBC in Japan [3]. They found that the 21-gene assay had stronger predictive power than tumor histological grade.

A study at tertiary referral centers in Tokyo and Saitama, Japan evaluated 104 women with ER+ ESBC, either without nodal involvement or with micrometastases, and showed that the 21-gene assay changed 33% of the adjuvant chemotherapy treatment recommendations, resulting in a net 19% absolute reduction in chemotherapy use [18]. Using the decision impact data from Japanese clinical practice, this study aimed to evaluate the cost-effectiveness of the 21-gene assay compared to traditional prognostic indicators for the guidance of adjuvant chemotherapy decisions in ER+, LN- ESBC patients from a societal perspective in Japan.

## Methods

This analysis considered Japanese women with ER+, LN- (including micrometastases) ESBC who were eligible for treatment with adjuvant chemotherapy after having undergone surgery for primary tumor removal and lymph node dissection.

### Analytical framework

**Figure 1:**
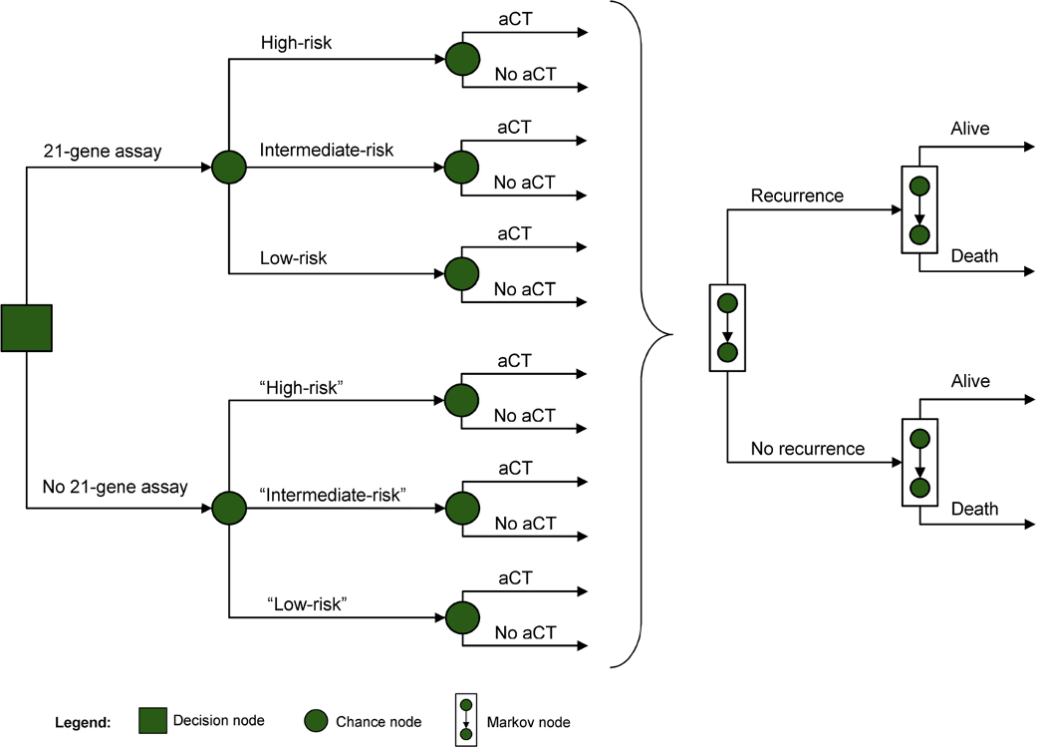
**Markov diagram.** The level of recurrence risk is based on the 21-gene assay. Quotation marks indicate values unknown to physicians and patients. Abbreviation: aCT, adjuvant chemotherapy.

Outcomes and costs were assessed from a Japanese societal perspective from surgery to death using a state-transition cohort (Markov) model (Figure 1). The primary comparator was clinical practice where risk assessment for 10-year distant recurrence was based on traditional clinicopathological factors recommended in guidelines [7]. The intervention was use of the 21-gene assay (Onco*type* DX® Breast Cancer Assay; Genomic Health, Inc., Redwood City, USA) to stratify patients into 3 risk groups – low, intermediate, or high. The probabilities of recommendations in favor of adjuvant chemotherapy before and after availability of the patients’ 21-gene assay results were obtained from the tertiary referral center study [18]. Thereafter, the model followed the incidence of distant recurrence, breast-cancer–related mortality, and non–breast-cancer–related mortality.

Direct medical costs were included for the 21-gene assay, adjuvant chemotherapy, surveillance (2 outpatient visits per year), adverse events and distant recurrence. Indirect costs included travel expenses, and patient time for travel and treatment. All costs are reported in 2013 Japanese Yen (JPY, ¥). Cost data from previous years were adjusted using consumer price indices for medical services and public transportation published by the Statistics Bureau of Japan [19], and wage indices published by the Japanese Ministry of Health, Labour and Welfare [20]. Summaries are provided selectively in United States Dollars (USD, $; conversion rate: 100 JPY = 1 USD) for comparison.

Costs and benefits of treatment were discounted at a 3% annual rate, which is in accordance with recommendations from the International Society for Pharmacoeconomics and Outcomes Research (ISPOR) [21] and has been used in prior cost-effectiveness studies in Japan [22, 23]. Inputs were varied individually in a one-way sensitivity analysis to evaluate the effects of each input variable’s uncertainty on the assay’s incremental cost-effectiveness ratio (cost per QALY gained).

### Data sources and assumptions

#### Impact of the 21-gene assay score on recommendations to use adjuvant chemotherapy

The tertiary referral centers study found that recommendations for adjuvant chemotherapy decreased by 19% overall as a result of using the 21-gene assay (Table 1) [18]. None of the women identified as having low distant recurrence risk were recommended to adjuvant chemotherapy after receiving their 21-gene assay results, down from 32% prior to testing. In the intermediate risk group, 19% fewer were recommended adjuvant chemotherapy, whereas recommendations increased by 18% in the high risk group.

**Table 1:** **Influence of the 21-gene assay on adjuvant chemotherapy recommendations**

Patient population*	Patients recommended by physicians to adjuvant chemotherapy, n (%)		
	Prior to assay	After assay	Change
All	48 (46.2)	28 (26.9)	−20 (−19.2)
Low-risk	16 (32.0)	0 (0.0)	−16 (−32.0)
Intermediate-risk	18 (48.6)	11 (29.7)	−7 (−18.9)
High-risk	14 (82.4)	17 (100.0)	3 (17.6)

*Risk group determined by the 21-gene assay. Risk groups: zero to 17 is low-risk, 18 to 30 is intermediate-risk, greater than 30 is high-risk.

Source: Yamauchi et al. Clin Breast Cancer 2013 [18].

#### Probability of recurrence, mortality, and adjuvant chemotherapy adverse events

Estimates of the baseline recurrence risk in Japan by the 21-gene assay’s risk groups were extracted from Toi et al., who studied the clinical validity of the assay in a Japanese population of 200 women with LN- ESBC (Table 2) [17]. These rates were lower than those previously reported for women from the US and UK [11, 14]. Data on the benefit of chemotherapy by 21-gene assay risk group were extracted from a separate validation study of patients from NSABP B-20 [15].

**Table 2:** **Model inputs**

Parameter	Mean	Sensitivity analysis		Source
		Low	High	
*Baseline recurrence risk by 21-gene assay risk group*				
Japan				
Low-risk	3.3%	1.1%	10.0%	[17]
Intermediate-risk	0.0%	0.0%	0.0%	[17]
High-risk	24.8%	15.7%	37.8%	[17]
US and UK				
Low-risk	5.4%	3.6%	8.5%	[11, 14]
Intermediate-risk	13.7%	8.6%	20.0%	[11, 14]
High-risk	29.2%	21.6%	37.2%	[11, 14]
*Relative reduction of recurrence with aCT by 21-gene assay risk group*				
Low-risk	0%	0%	54%	[15]
Intermediate-risk	39%	0%	76%	[15]
High-risk	74%	47%	87%	[15]
Costs*				
21-gene assay	¥350,000 ($3,500)	¥262,500	¥437,500	List price
Associated with aCT				
Drugs	¥561,813 ($5,618)	¥280,907	¥1,500,000	St. Luke’s Hospital (Tokyo, Japan)
Adverse events	¥170,831 ($1,708)	¥85,416	¥256,247	St. Luke’s Hospital (Tokyo, Japan), [24]
Patient time and transportation	¥68,500 ($685)	¥34,250	¥102,750	St. Luke’s Hospital (Tokyo, Japan)
Surveillance (2 visits/year)	¥25,416 ($254)	¥12,708	¥38,124	[23]
Per recurrence per year	¥2,405,924 ($24,059)	¥1,202,962	¥3,608,886	[22]
*Quality of life*				
No recurrence, no aCT	0.98	0.78	1.00	[23]
Progression	0.30	0.24	0.36	[26, 27]
QALY tariff of aCT	0.53	0.43	0.64	[23, 24, 28]
*Other assumptions*				
Age	49.8	35	75	St. Luke’s Hospital (Tokyo, Japan)
Annual mortality risk after progression	40%	20%	60%	[22, 23]
Time horizon, years	Lifetime			ISPOR guidelines

*Abbreviations:* *¥, JPY* Japanese Yen, *$, USD* United States Dollar, *aCT* adjuvant chemotherapy, *QALY* quality-adjusted–life-year, *ISPOR* International Society For Pharmacoeconomics and Outcomes Research.

A 3% time preference discount rate was applied in the basecase scenario (lower bound, 1%; upper bound, 5%) [21] *.*

*Per patient on average. Reported in 2013 currency. Conversion rate is 100 JPY per 1 USD.

Japanese-specific risks of grade 1, 2, or 3 adverse events were extracted from unpublished data from St. Luke’s Hospital analyzed by Tsugawa et al. The risk of fatal toxicity from adjuvant chemotherapy was extracted from Hillner and Smith [24]. The annual risk of dying from non–breast-cancer–related causes was based on population mortality rate data in Japan [25]. The annual risk of dying from breast-cancer–related causes after recurrence was extracted from previous models [22, 23].

#### Costs

The price of the 21-gene assay was set equal to the list price in Japan, (¥350,000). The cost of chemotherapy, ¥561,813, was based on data from one of the tertiary referral centers, St. Luke’s Hospital in Tokyo, Japan (Table 2). The total direct medical costs of salvage treatment and palliative care from the time of distant recurrence to time of death in Japan is ¥4,710,584 [22], representing an annual cost after recurrence equal to ¥2,405,924. Costs to patients and payers to manage adverse events were obtained from the tertiary referral center study and weighted by the proportion of patients who experienced the event. Additional indirect patient time and transportation costs were derived as the sum of lost time (valued based on average hourly wage in Japan) and transportation expenses (train fares) for visits related to adjuvant chemotherapy.

#### Quality of life

Health utility scores and quality-adjusted–life-year (QALY) tariffs were used to calculate QALYs. Health utility scores range from 0 for death to 1 for perfect health and quantify the desirability of a particular health state. QALY tariffs represent the reduction in QALYs caused by a procedure or event. These measures of patient preference and quality-of-life were extracted from published cost-effectiveness analyses in Japan and time-trade-off patient surveys (Table 2) [23, 26–28]. QALYs were computed as the lifetime sum of the product of each health state utility and mean time in that health state (adjusted for fixed annual discount rate).

### Data analysis

The changes in QALYs related to the immediate disutility of adjuvant chemotherapy and the later disutility of recurrence were analyzed. The lifetime costs and cost-effectiveness of the 21-gene assay versus current clinical practice were also reported. The main analysis employed estimates of distant recurrence risk from a Japanese validation study [17]. An alternative scenario was also evaluated with estimates of distant recurrence risk from US- and UK-based clinical validation studies [11, 14].

One-way sensitivity analyses were conducted to assess the robustness of the primary endpoint (cost per QALY gained). Each parameter was varied across its individual range to evaluate the effect of the parameters’ uncertainty on the results. The range for each parameter was extracted from 95% confidence intervals if reported, and broad ranges (±25% to ±50%) otherwise.

## Results

Reduction in adjuvant chemotherapy use (19%) due to the 21-gene assay led to immediate gains of 0.103 QALYs per patient (Table 3). The 21-gene assay increased adjuvant chemotherapy use and reduced distant recurrence risk among high-risk patients, resulting in an additional average later-term gain of 0.139 QALYs per patient, assuming baseline risk estimates from the Japanese validation study (Table 3a). In total, the 21-gene assay led to an average increase of 0.241 QALYs per patient.

**Table 3:** **Base-case results**

A. Baseline recurrence risk from a Japan-based validation study			
Main analysis	Without 21-gene assay	With 21-gene assay	Difference
Proportion receiving adjuvant chemotherapy	46.2%	26.9%	−19.2%
10-year recurrence-free survival, %	94.5%	95.0%	0.5%
QALYs			
Adjuvant chemotherapy (immediate)	−0.246	−0.144	0.103
Recurrence (long-term)	21.093	21.231	0.139
Total	20.847	21.088	0.241
Costs*			
21-gene assay		¥350,000	¥350,000
Acute costs			
Chemotherapy drugs	¥259,298	¥151,257	-¥108,041
Adverse events	¥78,845	¥45,993	-¥32,852
Patient time and transportation	¥31,615	¥18,442	-¥13,173
Monitoring costs until recurrence	¥520,493	¥524,238	¥3,744
Costs after recurrence	¥347,446	¥301,333	-¥46,113
Total	¥1,237,698	¥1,391,263	¥153,565
Cost per QALY gained		JPY	¥636,752
		USD	$6,368
**B. Baseline recurrence risk from US- and UK-based validation studies**			
**Alternative analysis**	**Without 21-gene assay**	**With 21-gene assay**	**Difference**
Proportion receiving adjuvant chemotherapy	46.2%	26.9%	−19.2%
10-year recurrence-free survival, %	89.4%	89.6%	0.3%
QALYs			
Adjuvant chemotherapy (immediate)	−0.246	−0.144	0.103
Recurrence (long-term)	19.533	19.590	0.057
Total	19.287	19.447	0.160
Costs*			
21-gene assay		¥350,000	¥350,000
Acute costs			
Chemotherapy drugs	¥259,298	¥151,257	-¥108,041
Adverse events	¥78,845	¥45,993	-¥32,852
Patient time and transportation	¥31,615	¥18,442	-¥13,173
Monitoring costs until recurrence	¥478,260	¥479,798	¥1,537
Costs after recurrence	¥899,695	¥882,593	-¥17,102
Total	¥1,747,715	¥1,928,084	¥180,369
Cost per QALY gained		JPY	¥1,129,442
		USD	$11,294

*Abbreviations: QALY* quality-adjusted–life-year, *¥, JPY* Japanese Yen, *$, USD* United States Dollar.

*Per patient on average. Reported in 2013 currency.

The 21-gene assay cost ¥350,000. Reductions in costs associated with adjuvant chemotherapy (−¥108,041), adverse events (−¥32,852), patient time and transportation (−¥13,173), and distant recurrence (−¥46,113) offset the testing cost. Delay and/or prevention of recurrence increased monitoring costs by ¥3,744. Total lifetime costs increased ¥153,565 per patient with the 21-gene assay, resulting in an incremental cost-effectiveness ratio of ¥636,752 ($6,368) per QALY gained.

In the alternative analysis, assuming the higher US and UK baseline distant recurrence risk estimates, the 21-gene assay increased QALYs by 0.160 years per patient on average (Table 3b). The QALY increase related to recurrence-free–survival improvement was smaller in this scenario (0.057) due to the greater likelihood of recurrence.

Lifetime costs in this alternative scenario increased by ¥180,369 per patient on average with the 21-gene assay. Monitoring costs increased by ¥1,537, while the costs to manage distant recurrence decreased by ¥17,102. Other costs were identical to the main analysis. The higher US and UK recurrence risks lead to less recurrence-related savings and a smaller increase in monitoring costs, resulting in a greater increase in lifetime costs compared to the main analysis. The cost per QALY gained in the alternative scenario was ¥1,129,442 ($11,294).

**Figure 2:**
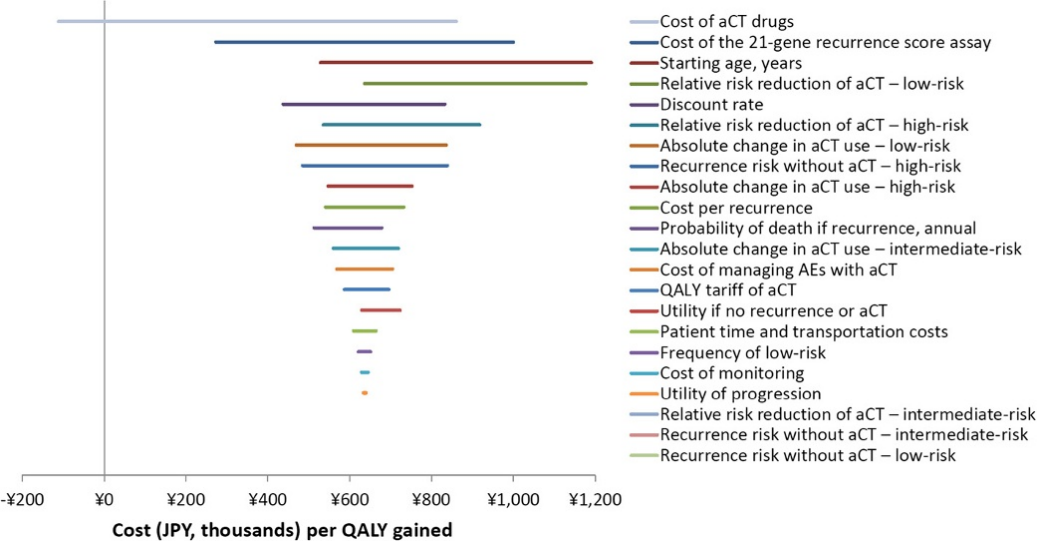
**One-way sensitivity analysis tornado diagram: baseline recurrence risk from a Japan-based validation study.** Abbreviations: aCT, adjuvant chemotherapy; AEs, adverse events; QALY, quality-adjusted–life-year; ¥, JPY, Japanese Yen in 2013 currency.

With Japanese distant recurrence risk data, the parameters whose change or uncertainty most altered the cost per QALY gained in the one-way sensitivity analysis were the: (1) cost of chemotherapy drugs, (2) cost of the 21-gene assay, and (3) patient’s age at diagnosis (Figure 2). The maximum cost per QALY gained was ¥1,189,962 ($11,900), which resulted only if the average age of diagnosis rose from 49.8 to 75. The next highest cost per QALY gained, ¥1,177,069 ($11,771), resulted only if the relative distant recurrence risk reduction due to chemotherapy in low-risk patients (the fourth most influential variable) was increased from the base-case value of zero to 54%. These were the only two scenarios under which the projected cost per QALY gained was above ¥1,000,000.

**Figure 3:**
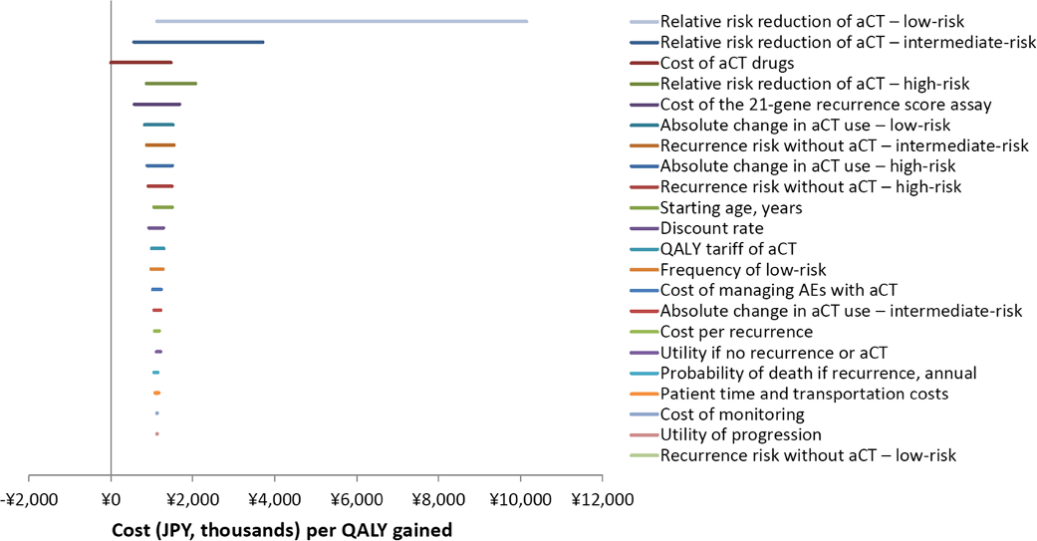
**One-way sensitivity analysis tornado diagram: baseline recurrence risk from a US- and UK-based validation studies.** Abbreviations: aCT, adjuvant chemotherapy; AEs, adverse events; QALY, quality-adjusted–life-year; ¥, JPY, Japanese Yen in 2013 currency.

Given US- and UK-based distant recurrence risk data, the parameters whose change or uncertainty most altered the cost per QALY gained were the: (1) relative distant recurrence risk reduction due to chemotherapy in low-risk patients and (2) in intermediate-risk patients, and (3) cost of chemotherapy drugs (Figure 3). Once again, increasing the relative distant recurrence risk reduction due to chemotherapy in low-risk patients from zero to 54% results in the highest cost per QALY gained. This scenario’s cost per QALY gained was unusually high at ¥10,142,498 ($101,425); the next highest cost per QALY gained was ¥3,703,793 ($37,038), which resulted when the relative distant recurrence risk reduction due to chemotherapy in intermediate risk patients was increased from 39% to 76%.

Cost-savings were predicted with the 21-gene assay when adjuvant chemotherapy drug costs increased beyond ¥1,360,351 ($13,604) given Japanese distant recurrence risk data. With US- and UK-based distant recurrence data, cost-savings were predicted when adjuvant chemotherapy drug costs exceeded ¥1,499,733 ($14,997). These values were comparable to the drug costs of some regimens containing prophylactic granulocyte colony-stimulating factor (G-CSF) reported by Ishiguro et al. in 2010 [22].

## Discussion

Using actual chemotherapy recommendations in a Japanese setting, this study found that the use of the 21-gene assay resulted in an increase of 0.241 QALYs and increased lifetime costs of ¥153,565 per patient on average. The consequent cost per QALY gained was ¥636,752 ($6,368).

Previously, 21-gene assay recommendations were compared with St. Gallen guideline criteria in a societal cost-effectiveness analysis by Kondo et al. [23]. Guidelines have been used as surrogate decision impact parameters in cost-effectiveness studies of diagnostics early in their development or diffusion when use is fairly limited [29]. As clinical decision impact data becomes available, it is important to validate those analyses that used surrogate parameters, as has been done for the 21-gene assay in other settings [30, 31]. The study herein built upon previous work to provide the first available societal cost-effectiveness estimate in Japan, applying actual recommendations for chemotherapy from a tertiary referral center. This study included direct chemotherapy- and distant recurrence-related costs, and indirect costs associated with time dedicated to treatment and transportation fees.

The one-way sensitivity analysis showed that the cost per QALY gained was less than ¥1,189,962 ($11,900) across all ranges of parameters. The cost-effectiveness ratio was highest when patient age at diagnosis was high or chemotherapy was assumed to be extremely beneficial for low-risk patients. The 95% confidence interval for the effect of chemotherapy in low-risk patients was very wide, and 84% of that range indicated no benefit to chemotherapy for low-risk patients [15].

The largest change in the cost per QALY gained in the one-way sensitivity analysis resulted when the cost of chemotherapy drugs was varied. This finding is especially relevant as third-generation regimens using G-CSF have recently been reported to have a cost-effectiveness ratio lower than the commonly accepted threshold value (¥6 million per QALY gained) [22]. If adjuvant chemotherapy drug costs increase to the values previously reported for G-CSF–containing chemotherapy regimens in Japan, this model shows that the 21-gene assay would be cost-saving, regardless of the distant recurrence risk data source.

The 21-gene assay validation study in Japan showed no distant recurrences in the intermediate-risk group [17]. Therefore, the benefit of chemotherapy for the intermediate group had no effect on the cost or QALY results in the main analysis. By contrast, US- and UK-based validation studies showed higher distant recurrence risks overall, increasing from the low- to intermediate- to high-risk groups. When estimates from the US- and UK-based studies were applied, varying the benefit (relative risk reduction) due to chemotherapy in the intermediate-risk group in the one-way sensitivity analysis led to the second-largest change in the cost per QALY gained result.

Applying real chemotherapy recommendation changes, this analysis’ findings differed from those of a previous analysis by Kondo et al. in several significant respects [23]. Total QALYs were slightly higher in this study (20.8 years without and 21.1 years with the 21-gene assay) than in Kondo et al. (19.5 years without and 20.1 years with); however, the QALYs gained in this study (0.241) was less than half of that reported by Kondo et al. (0.63). In this analysis, the lifetime costs do not exceed ¥2 million in any of the scenarios tested regardless of 21-gene assay use, whereas Kondo et al. reported costs exceeding ¥3 million. Using US- and UK-based recurrence risk estimates lowered the total QALYs results. In this analysis, total QALYs was 19.3 years without and 19.4 years with the 21-gene assay, slightly below those reported by Kondo et al. The 0.160 QALY gain projected here is approximately one-fourth of the gain estimated by Kondo et al. Factors that may have led to the differences include (1) a larger decrease in recommendations in favor of adjuvant chemotherapy found in actual clinical practice (19% herein versus 8% when applying St. Gallen criteria) and (2) a target population selection that excluded patients with HER2+ tumors who would presumably receive trastuzumab regardless of the 21-gene assay result. Given the unexplained differences among these model findings, it may be a useful exercise to directly and collaboratively cross-validate the available models, as has been performed in other fields [32].

Several limitations are important to consider when interpreting the results of this study. First, the decision impact study at the tertiary center excluded women (1) whose recurrence risk was assessed by a physician as very low, (2) who had already chosen to undergo chemotherapy, or (3) who could not afford to participate. Therefore, the proportion of patients whose treatment recommendations are altered by the 21-gene assay may be different if the assay were used without consideration of these exclusion criteria. Second, utilities estimates from Japanese women are not available. For more than two decades, it has been well-established that attitudes about cancer and its treatments may differ across countries [33]. It would be relevant in the future to have information from studies on patients’ experiences in Japan, especially utility data. Finally, the 21-gene assay has recently been shown to also predict local recurrence [13]. Excluding this outcome benefit predictably underestimates the benefits to patients and cost-savings to society. This analysis likely overestimated the cost per QALY gained with the 21-gene assay compared to clinical practice using traditional clinicopathological factors.

## Conclusions

Previous analyses concluded that the 21-gene assay “is indicated as cost-effective in Japan” when compared to clinical guidelines [23]. This new analysis, which incorporated clinical recommendations made in Japanese tertiary referral centers, supports this finding.

## Authors’ information

JH is a member of the Ethics Committee for the American Society of Clinical Oncology, co-chair of the Health Science Task Force on Communicating Health Economics Research in the International Society for Pharmacoeconomics and Outcomes Research (ISPOR), and co-editor of the *Value in Health* journal published on behalf of ISPOR.

## Abbreviations

ER+: Estrogen-receptor–positive
LN-: Lymph-node–negative
ESBC: Early-stage breast cancer
NCCN: National Comprehensive Cancer Network
ISPOR: International Society for Pharmacoeconomics and Outcomes Research
QALY: Quality-adjusted–life-year
JPY: Japanese Yen
USD: United States Dollar
aCT: Adjuvant chemotherapy
G-CSF: Granulocyte colony-stimulating factor.
